# Abstract of Meteorological Observations for May, 1855, Made at Philadelphia, Pa.

**Published:** 1855-07

**Authors:** James A. Kirkpatrick


*Abstract of Meteorological Observations for May,* 1855, *made at Philadelphia, Pa. Latitude* 39° 57′ 28″ *N., Longitude* 75° 10′ 40″ *W. from Greenwich.* By PROF. JAMES A. KIRKPATRICK.

| 1855. May. | BAROMETER. | | THERMOM. | | | | | | | |
|---|---|---|---|---|---|---|---|---|---|---|
| | Daily Mean | Mean Daily Range. | Daily Mean | Mean Daily Range | Dew Point 2 P.M. | Force of Vapor 2 P.M. | Rel. Humid. 2 P.M. | Rain. | Prevailing Winds. | Remarks. |
| | Inches. | Inches. | Deg. | Deg. | Deg. | Inches. | Hunds. | Inch. | Points. | |
| 1 | 29.781 | .143 | 59.8 | 6.3 | 58.0 | .499 | .91 | 0.030 | NW. | M. *Rain*; aft. and ev. cloudy. |
| 2 | 29.963 | .182 | 61.7 | 4.5 | 43.3 | .335 | .47 | | (Var.) | Cloudy. |
| 3 | 29.900 | .030 | 54.7 | 7.0 | 43.3 | .295 | .68 | 0.005 | NE. | Cloudy; ev. *Rain.* |
| 4 | 29.733 | .200 | 58.7 | 5.3 | 27.3 | .136 | .21 | | NW. | Clear. |
| 5 | 29.765 | .032 | 62.0 | 3.3 | 25.3 | .124 | .16 | | NW. | Clear. |
| 6 | 29.706 | .073 | 65.3 | 3.3 | 46.0 | .389 | .45 | | (Var.) | Clear; m. hazy. |
| 7 | 29.634 | .072 | 59.0 | 6.3 | 30.7 | .177 | .26 | | E. | Cloudy; m. hazy. [*l'st* 29.449 |
| 8 | 29.531 | .106 | 48.0 | 11.0 | 43.0 | .284 | .78 | 0.238 | (Var.) | Cl'y; m. aft. & n't *Rain. Bar.* |
| 9 | 29.774 | .243 | 45.3 | 2.7 | 38.3 | .235 | .64 | 0.546 | NW. | Cl'dy; m. *Rain; Th. lo'st.* 40° |
| 10 | 29.941 | .170 | 56.0 | 10.7 | 31.7 | .176 | .32 | | WNW. | Clear; m. hazy. |
| 11 | 29.878 | .063 | 62.0 | 6.0 | 33.7 | .221 | .29 | | W. | Clear. |
| 12 | 29.847 | .052 | 63.3 | 1.3 | 39.3 | .296 | .38 | | WSW. | Cloudy. |
| 13 | 29.855 | .015 | 65.3 | 2.0 | 46.3 | .390 | .50 | | (Var.) | M. clear; aft. and ev. cloudy. |
| 14 | 29.810 | .046 | 72.8 | 7.5 | 32.3 | .268 | .21 | | SW. | Cloudy. |
| 15 | 29.815 | .036 | 76.3 | 3.8 | 45.0 | .467 | .37 | | SW. | Cloudy; *Therm. highest* 87°. |
| 16 | 29.703 | .112 | 77.5 | 3.2 | 39.3 | .372 | .30 | | SW. | Cloudy. |
| 17 | 29.833 | .137 | 65.5 | 4.8 | 43.3 | .335 | .47 | 0.482 | NW. | M. *Rain;* aft. & ev. clear. |
| 18 | 29.952 | .119 | 63.3 | 4.7 | 35.7 | .252 | .31 | | NE. | Cloudy. |
| 19 | 29.637 | .315 | 55.8 | 7.7 | 59.0 | .500 | 1.00 | 1.543 | NE. | Cloudy; *Rain* all day. |
| 20 | 29.504 | .133 | 61.5 | 5.7 | 48.7 | .389 | .63 | 0.035 | NW. | M. driz'ng rain; a. cl'y; ev. cl'r |
| 21 | 29.576 | .071 | 63.7 | 6.8 | 32.3 | .207 | .27 | | NW. | M. cloudy; aft. and ev. clear. |
| 22 | 29.685 | .106 | 62.2 | 1.5 | 34.7 | .237 | .30 | | NW. | Clear. |
| 23 | 29.782 | .097 | 64.5 | 3.3 | 44.7 | .382 | .44 | | (Var.) | Clear. [*th.* $9\frac{1}{2}$; *R.* from 10 P.M. |
| 24 | 29.785 | .017 | 74.5 | 10.3 | 60.7 | .704 | .59 | 0.154 | (Var.) | *R.* 4 to 6 A.M.; *l'ng* from $7\frac{1}{2}$ P.M. |
| 25 | 29.820 | .035 | 71.0 | 7.8 | 37.3 | .292 | .31 | | N. | M. and aft. cloudy; ev. clear. |
| 26 | 29.875 | .056 | 64.3 | 6.7 | 34.0 | ·219 | .30 | | NNW. | M. and aft. cloudy; ev. clear. |
| 27 | 29.874 | .010 | 63.7 | 4.0 | 33.7 | .221 | .29 | | NW. | Clear. |
| 28 | 29.921 | .047 | 67.7 | 4.0 | 38.7 | .273 | .31 | | (Var.) | Cloudy. |
| 29 | 29.997 | .073 | 69.7 | 3.3 | 40.3 | .349 | .34 | | (Var.) | Cloudy. [*Bar. hig'st* 30.124 |
| 30 | 30.095 | .098 | 68.7 | 1.3 | 46.3 | .430 | .43 | | S. | M. and aft. cloudy; ev. clear; |
| 31 | 30.044 | .051 | 72.8 | 4.2 | 50.7 | .510 | .48 | | S. | M. clear; aft. and ev. cloudy. |
| Means for May, 1855 | 29.807 | .095 | 63.8 | 5.2 | 41.4 | .321 | .43 | 3.033 | N. 48° W., 56–100. | |
| 4 yrs | 29.858 | .093 | 63.8 | 5.2 | 47.1 | | | 4.635 | N. 73° W., 37–100. | |

The Monthly Range of the Barometer was 0.675 of an inch., and of the Thermometer 47°.

